# Association of lipid lowering drugs and the risk of systemic lupus erythematosus: a drug target Mendelian randomization

**DOI:** 10.3389/fphar.2023.1258018

**Published:** 2023-10-30

**Authors:** Tong Wu, Ling Ye, Shenglan Wang, Jie Huang, Jing Zhang

**Affiliations:** ^1^ Department of Rheumatology and Immunology, Sichuan Provincial People’s Hospital, School of Medicine, University of Electronic Science and Technology of China, Chengdu, China; ^2^ Department of Neurology, Affiliated Hospital of Chengdu University, Chengdu, China

**Keywords:** lipid lowering drugs, systemic lupus erythematosus, risk, drug target, Mendelian randomization

## Abstract

**Background and objective:** An interaction between low-density lipoprotein level, lipid-lowering drugs, and systemic lupus erythematosus (SLE) was reported by previous studies. However, whether lipid-lowering drugs provided protective effect for reducing the risk of SLE was unclear. We aimed to clarify this causal relationship through a drug-target Mendelian randomization (MR) study.

**Methods:** Genetic instruments—single nucleotide polymorphism (SNPs)—were utilized to proxy inhibition of the three genes—3-hydroxy-3-methylglutaryl-CoA reductase (HMGCR), proprotein convertase subtilisin/kexin type 9 (PCSK9), and Niemann-Pick C1-Like 1(NPC1L1), which was corresponded to three lipid-lowering drugs—statins, evolocumab, and ezetimibe. Low-density lipoprotein (LDL) cholesterol was selected as the biomarker for the measurement of the inhibitors of HMGCR, PCSK9, and NPC1L1, and the genetic data were acquired from the Global Lipids Genetics Consortium, which consisted of 1.3 million participants of European ancestry and 146.5 thousand participants of East Asian ancestry. The genetic dataset of SLE was acquired from two large-scale GWAS studies; one recruited 23,210 participants (7,219 SLE cases and 15,991 controls) of European ancestry and the other one recruited 12,653 participants (4,222 SLE cases and 8,431 controls) of Chinese ancestry. The primary analysis used the inverse variance weighted (IVW) method. Four additional sensitivity analyses, colocalization analysis, and stratification analysis were performed.

**Results:** The primary analysis showed that inhibition of PCSK9 (evolocumab) was associated with a significantly lower risk of SLE [odds ratio (OR) 0.51, 95%CI 0.34 to 0.76, *p* = 0.001] in the European population. The secondary analyses had similar findings. Stratification analysis demonstrated that the preventive effect of PCSK9 inhibition for SLE was similar in both males and females. However, the results were not replicated in the East Asian population. The inhibition of HMGCR (statins) and NPC1L1 (ezetimibe) did not cause a lower risk of SLE.

**Conclusion:** Evolocumab might provide a protective effect on the risk of SLE in the European population, but statins and ezetimibe might not have the protective effect. Further research is necessary to elucidate the specific mechanisms and potential therapeutic applications of PCSK9 inhibitors (evolocumab) in the context of SLE protection.

## Introduction

Systemic lupus erythematosus (SLE) is a disease characterized by abnormal immune system activity resulting in a variety of clinical presentations and manifestations, including lesions of the skin, kidneys, joints, and nervous system. The global prevalence of SLE ranges from 30 to 150 per 100,000 and has been increasing over the past 20 years (Durcan et al., 2019). SLE usually occurs in women of childbearing age. However, about 10%–20% of cases have an onset after the age of 50 (Fettig et al., 2013).

Treatment of SLE remains unsatisfactory. The standardized mortality for SLE is estimated to be 2.4%–5.9%, with a 10-year survival rate exceeding 90%. Premature mortality is mainly due to lupus nephritis and cardiovascular disease (CVD). Although the use of corticosteroids, immunosuppressants and immunomodulators has significantly improved the life expectancy of SLE patients, the increased risk of CVD and CVD-related mortality implies an unmet treatment need in SLE patients. Hydroxychloroquine is an immunomodulator used in the treatment of SLE and has been shown to be effective in reducing serum low-density lipoprotein (LDL) cholesterol levels in patients with SLE (Babary et al., 2018), which implied an interaction between aberrant immune activation and dysfunction of lipid metabolism. There are studies reporting that higher proprotein convertase subtilisin/kexin type 9 (PCSK9, the gene associated with high LDL levels) levels correlate with SLE severity (Liu et al., 2020; Sánchez-Pérez et al., 2020), suggesting that blocking the PCSK9 function would be protective of SLE and its CVD risk. However, atorvastatin, a lipid-lowering drug developed by blocking HMGCR gene function, was found to have no protective effect on the progression of subclinical atherosclerosis in SLE patients (Schanberg et al., 2012). The diverse effects of PCSK9 and HMGCR inhibitors on SLE raised a relevant question: do lipid-lowering drugs have a protective effect against SLE? Conducting a randomized controlled trial (RCT) to answer this question would be time consuming and costly.

Drug-target Mendelian randomization (MR) is a novel trial design that uses genetic tools—single nucleotide polymorphisms (SNPs)—to predict the clinical outcome of blocking a specific gene function (e.g., the PCSK9 gene). Several drug-target MR studies have successfully predicted the effect of lipid-lowering drugs on psoriasis, epilepsy, and non-alcoholic fatty liver disease (Li et al., 2023; Liang et al., 2023; Zhao et al., 2023). These studies promoted drug repurposing and saved resources for medical research. For these reasons, we conducted a drug-target MR study to evaluate the protective effect of different lipid-lowering drugs on SLE risk.

## Materials and methods

### Study overview

**FIGURE 1:**
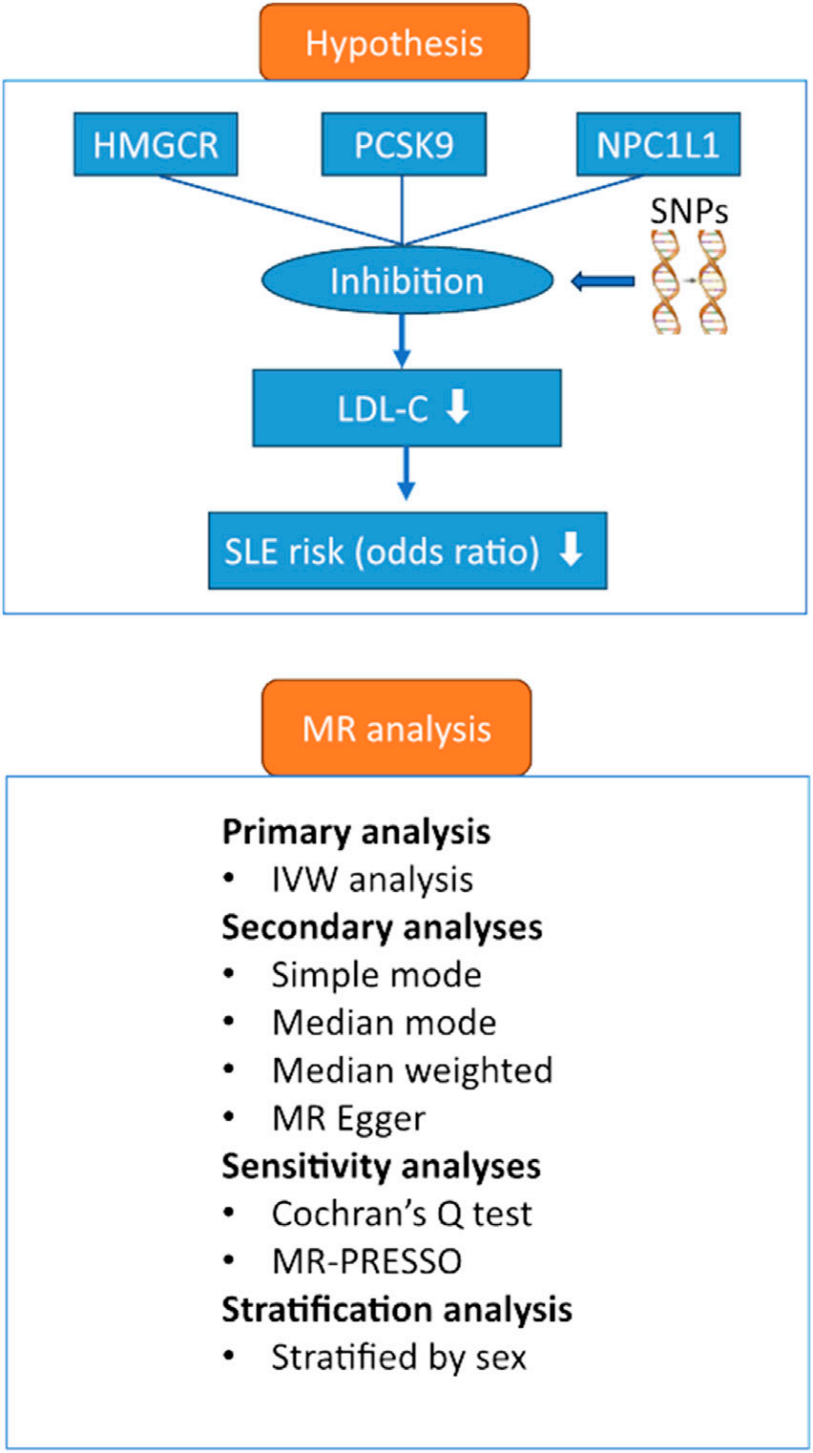
Hypothesis and study design. Abbreviations: HMGCR, 3-hydroxy-3-methylglutaryl-CoA reductase; IVW, inverse variance weighted; SNPs, single nucleotide polymorphisms; LDL-C, low-density lipoprotein cholesterol; PCSK9, proprotein convertase subtilisin/kexin type 9 serine protease; MR, Mendelian randomization; MR-PRESSO, Mendelian Randomization Pleiotropy RESidual Sum and Outlier; NPC1L1, Niemann-Pick C1-Like 1; SLE, systemic lupus erythematosus.

The primary objective of this study was to examine whether inhibition of three lipid-lowering drug targets-HMGCR, PCSK9, and NPC1L1-was associated with a lower risk of SLE, and the secondary objective was to examine whether inhibition differed between women and men. The study used a two-sample MR design in which we selected genetic tools that predicted inhibition of HMGCR, PCSK9, and NPC1L1 (as measured by a significant reduction in LDL-C) in one study population and examined whether these tools caused a reduction in risk in another population. The study was designed and conducted in accordance with the Strengthening the Reporting of Observational Studies in Epidemiology Using Mendelian Randomization (STROBE-MR) statement (Skrivankova et al., 2021). Ethical approvals and informed consent were obtained for the original GWAS studies; we used publicly available genetic data at the summary level, and no additional ethical approvals were required. Figure 1 shows the hypothesis and study design.

### Data source

Low-density lipoprotein (LDL) cholesterol was selected as the biomarker to measure inhibitors of HMGCR, PCSK9 and NPC1L1. The LDL genetic data set was selected from the Global Lipids Genetics Consortium, the largest GWAS meta-analysis of lipids to date (Graham et al., 2021). This dataset consisted of 1.3 million participants of European ancestry and 146.5 thousand participants of East Asian ancestry. We also acquired data stratified by sex, aiming to determine whether sex had an impact on the effect of the inhibitors of HMGCR, PCSK9, and NPC1L1 on the risk of SLE.

The genetic dataset of SLE was acquired from two large-scale GWAS studies; one recruited 23,210 participants (7,219 SLE cases and 15,991 controls) of European ancestry (Bentham et al., 2015) and the other one recruited 12,653 participants (4,222 SLE cases and 8,431 controls) of Chinese ancestry (Wang et al., 2021). The participants of European ancestry were diagnosed according to the American College of Rheumatology classification criteria (Bentham et al., 2015). The medical records of the participants of Chinese ancestry were reviewed to validate that all subjects met the revised criteria of the American College of Rheumatology for SLE diagnoses (Yang et al., 2010).

### Selection of genetic instruments

We adopted a selection strategy similar to previous studies examining the effect of lipid-lowering drugs on other diseases (Liang et al., 2023; Zhao et al., 2023), which included three steps. First, we selected SNPs associated with LDL at genome-wide significance (a *p*-value cut-off point of 5 × 10^−8^). Second, we performed linkage disequilibrium (LD) clumping (*r* ^2^ < 0.01 and clump window within 10,000 kilobases) to minimize the probability of genetically linked SNPs. Third, we screened the remaining SNPs for an inverse association with LDL levels within ±100 kb of the HMGCR gene (assembly in GRCh37.p13: chromosome 5: 74,630,496–74,660,434), the PCSK9 gene (assembly in GRCh37.p13: chromosome 1: 55,037,016–55,067,382), and NPC1L1 gene (assembly in GRCh37.p13: chromosome 7: 44,549,252–44,583,807) to instrument statins, ezetimibe, and evolocumab, respectively. All effect estimates were scaled up from the individual SNP-level effects on lipid levels to reflect the equivalent of a 1-mmol/L (LDL cholesterol for 38.7 mg/dL) change in lipid levels.

### Statistical analysis

The primary analysis of our study was the application of inverse variance weighted (IVW) analysis to obtain an overall effect of the selected SNPs predicting inhibition of the target genes on the risk of SLE. The effect estimate (*β*) was calculated by dividing the effect of each SNP predicting the risk of SLE by the effect of the SNP predicting the inhibition of HMGCR, PCSK9, and NPC1L1, and the estimates were pooled using the IVW method. To examine the presence of weak instrumental bias, we calculated the F-statistic value by dividing the square of the *β* by the square of its corresponding standard error (se), and an F-statistic >10 was a sign of adequate instrumental strength (Burgess et al., 2011).

The first assumption of MR required a significant association of genetic instruments with exposure (normally defined as a *p* < 5 × 10^−8^), and the second assumption required that the genetic instruments had no common cause with the outcome. In the process of SNP selection, we applied LD clumping to ensure the second assumption was not violated. LD clumping was performed by setting the r2 threshold as 0.01 and the clump window within 10,000 kilobases. We additionally performed a Bayesian colocalization analysis to estimate the possibility of the selected SNPs affecting SLE through non-lipid pathways. Default prior probability settings were adopted, and the calculation was performed by using the R package coloc 5.2.2.

The third assumption should influence the outcome only through the exposure. To establish this assumption, we applied the MR-Egger regression model to detect any directional pleiotropy and provide a consistent estimate of the causal effect under a weaker assumption—the InSIDE (Instrument Strength Independent of Direct Effect) assumption (Burgess and Thompson, 2017). We also used MR-PRESSO (Mendelian Randomization Pleiotropy RESidual Sum and Outlier) to detect horizontal pleiotropy, and if the pleiotropy existed, we applied outlier adjustment to the effect estimates. The simple mode, weighted mode, and weighted median analyses were also applied to test whether the results were consistent with the primary analysis.

Multivariate MR analysis was performed to determine whether lifestyle factors, dietary factors, and exercise played important roles in the causal effect of lipid-lowering drugs on SLE risk. According to previous reviews on SLE risk (Vordenbäumen et al., 2023; Xiao et al., 2023), we identified smoking status and alcohol intake frequency as the first group of lifestyle factors, selenium and iron intake as the second group of dietary factors, and accelerometer-based physical activity measurement as the third group of physical activity-related factors. Data for the three groups of factors were obtained from the UK Biobank.

All analyses were performed in the R environment (version 4.2.2) with the *TwoSampleMR* package.

## Results

Five SNPs were selected to proxy inhibition of the HMGCR gene, which had F-statistics ranging from 38.7 to 349.1 (mean F-statistic of 128.6) and providing sizes of reduction of LDL cholesterol from −0.038 to −0.073 mmol/L. Eleven SNPs were selected to proxy inhibition of the PCSK9 gene, having F-statistics from 34.7 to 650 (mean F-statistics of 172) and generating reduction sizes of LDL cholesterol from −0.039 to −0.089 mmol/L. Three SNPs were included to proxy inhibition of the NPC1L1 gene, having F-statistics from 41 to 90 (mean F-statistic of 71.2) and leading to reductions of LDL cholesterol from −0.036 to −0.049 mmol/L. The other information on the selected SNPs was shown in Supplementary Table S1.

**FIGURE 2:**
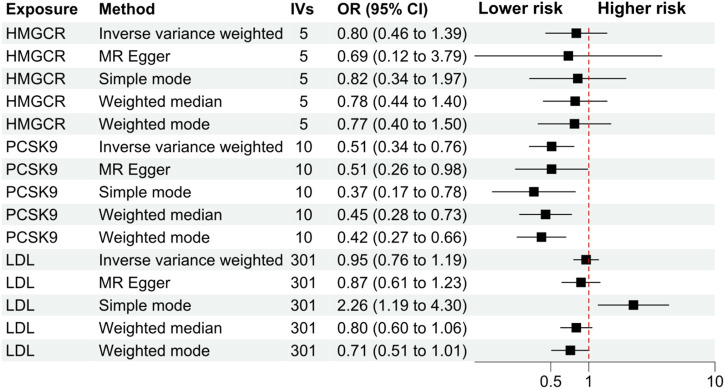
Secondary analyses. Abbreviations: HMGCR, 3-hydroxy-3-methylglutaryl-CoA reductase; IVs, number of instrumental variables; LDL, low-density lipoprotein cholesterol; PCSK9, proprotein convertase subtilisin/kexin type 9 serine protease; OR, odds ratio; SLE, systemic lupus erythematosus. Footnotes: The analysis of NPC1L1 (Niemann-Pick C1-Like 1) inhibition was not included in the secondary analyses, since only two SNPs passed the data harmonization process and the number of SNPs was insufficient for secondary analyses.

**FIGURE 3:**
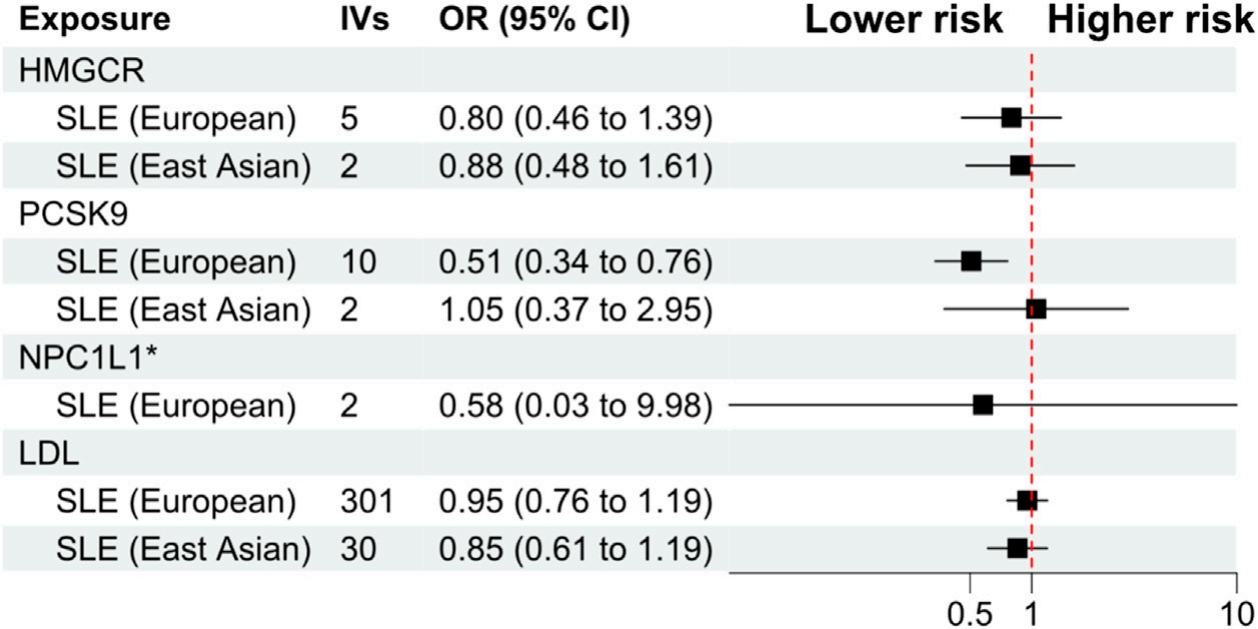
Primary analysis. Abbreviations: HMGCR, 3-hydroxy-3-methylglutaryl-CoA reductase; IVs, number of instrumental variables; LDL, low-density lipoprotein cholesterol; NPC1L1, Niemann-Pick C1-Like 1; PCSK9, proprotein convertase subtilisin/kexin type 9 serine protease; OR, odds ratio; SLE, systemic lupus erythematosus.

**FIGURE 4:**
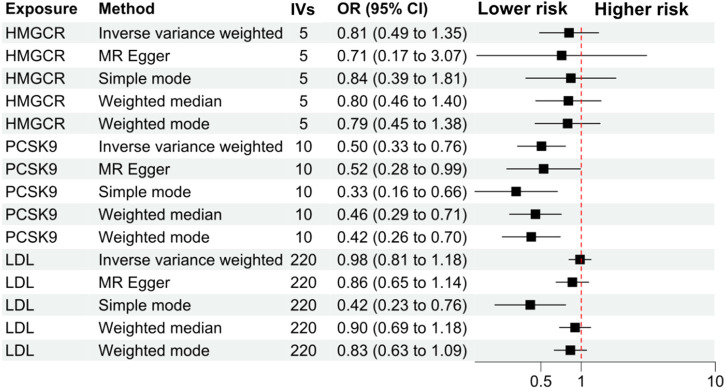
Analyses stratified by female gender. Abbreviations: HMGCR, 3-hydroxy-3-methylglutaryl-CoA reductase; IVs, number of instrumental variables; LDL, low-density lipoprotein cholesterol; NPC1L1, Niemann-Pick C1-Like 1. PCSK9, proprotein convertase subtilisin/kexin type 9 serine protease; OR, odds ratio; SLE, systemic lupus erythematosus. Footnotes: The analysis of NPC1L1 (Niemann-Pick C1-Like 1) inhibition was not included in the stratification analysis, since only two SNPs passed the data harmonization process and the number of SNPs was insufficient for stratification analyses.

**FIGURE 5:**
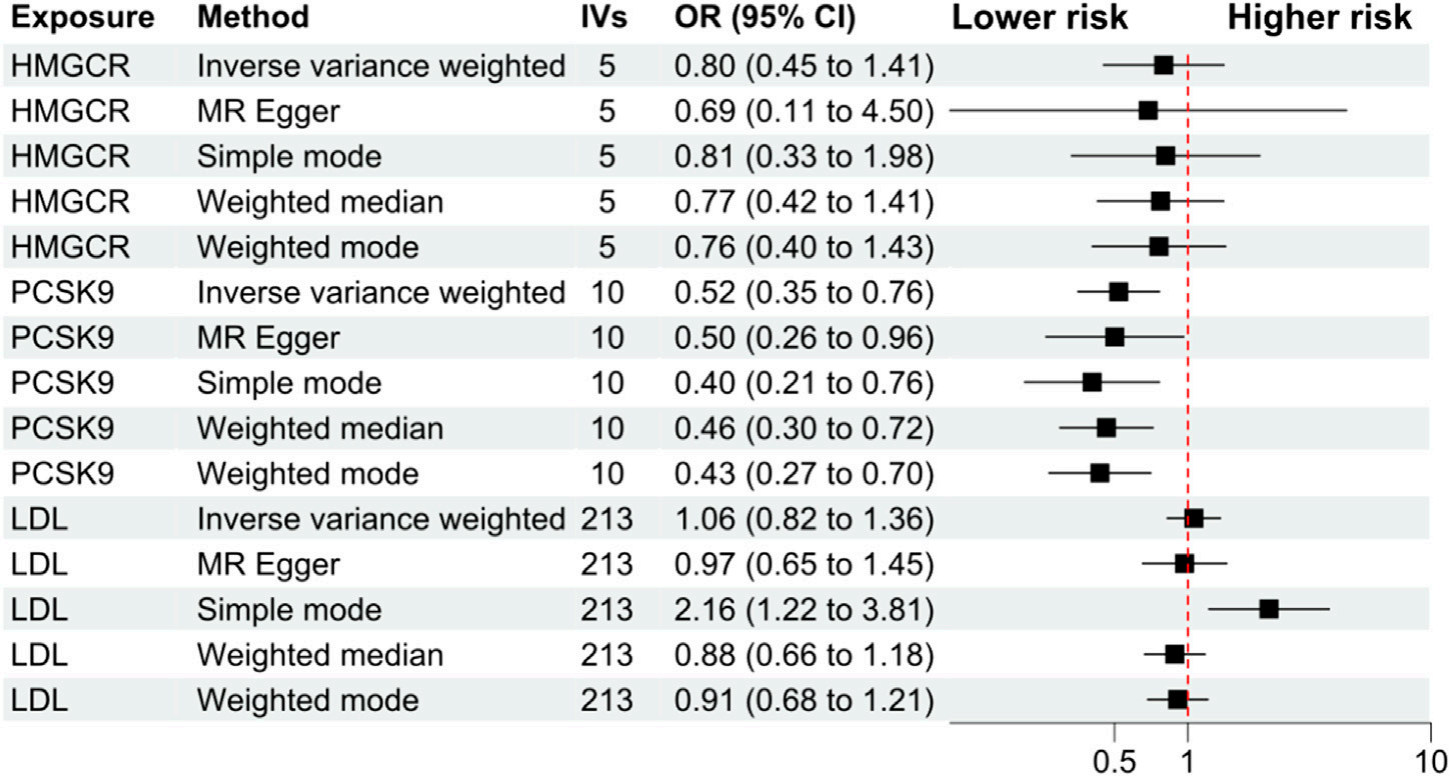
Analyses stratified by male gender. Abbreviations: HMGCR, 3-hydroxy-3-methylglutaryl-CoA reductase; IVs, number of instrumental variables; LDL, low-density lipoprotein cholesterol; NPC1L1, Niemann-Pick C1-Like 1; PCSK9, proprotein convertase subtilisin/kexin type 9 serine protease; OR, odds ratio; SLE, systemic lupus erythematosus. Footnotes: The analysis of NPC1L1 (Niemann-Pick C1-Like 1) inhibition was not included in the stratification analysis, since only two SNPs passed the data harmonization process and the number of SNPs was insufficient for stratification analyses.

The primary analysis showed that inhibition of PCSK9 was associated with a significantly lower risk of SLE [odds ratio (OR) 0.51, 95%CI 0.34 to 0.76, *p* = 0.001] in the European population. The secondary analyses—simple mode, weighted mode, weighted median, and MR Egger analysis showed similar findings (Figure 2; Supplementary Table S2). However, PCSK9 inhibition in the East Asian population was not associated with a lower risk of SLE (Figure 3). The inhibition of HMGCR and NPC1L1 did not cause a lower risk of SLE in both European and East Asian populations (Figure 3). Stratification analysis showed similar effect in females and males (Figures 4, 5; Supplementary Table S3). The MR Egger regression analysis showed no evidence of pleiotropic effect, with the intercept value being close to zero and *p*-values greater than 0.05 (Supplementary Table S4). The MR-PRESSO analysis did not detect any horizontal pleiotropic effect, and no significant heterogeneity was found in the analyses.

The colocalization analysis showed that the posterior probability of colocalization between LDL and SLE in the HMGCR, PCSK9, and NPC1L1 gene regions was 1.65%, 50.4%, and 2.23% on the presence of a causal variant for the outcome, respectively, using the European population. The sensitivity analysis of the colocalization analysis with posterior probability >0.9 revealed no common causal pathways for LDL levels in the three gene regions and SLE (Supplementary Figures S1–S3).

Table 1 shows the results of the multivariate MR analysis. The analysis showed that smoking status and alcohol consumption frequency were associated with SLE risk; the two lifestyle factors reduced the protective effect of PCSK9 inhibition. Selenium intake was positively associated with SLE risk, whereas iron intake was negatively associated with SLE risk; multivariate analysis showed that iron intake mediated part of the PCSK9 inhibitory effect. Physical activity was inversely associated with SLE risk, which also mediated part of the PCSK9 inhibitory effect.

**TABLE 1:** Multivariate Mendelian analysis.

Variables	HMGCR		PCSK9	
	Beta (se)	*p*-value	Beta (se)	*p*-value
Group 1				
Smoking status	4.47 (5.04)	0.469	0.51 (10.97)	0.965
Alcohol intake frequency	1.16 (1.94)	0.608	0.7 (2.26)	0.767
Lipid lowering	−0.45 (0.24)	0.208	−0.6 (0.38)	0.166
Group 2				
Selenium	25.1 (45.22)	0.635	8.22 (43.48)	0.856
Iron	−72.97 (57.12)	0.33	−2.3 (18.18)	0.904
Lipid lowering	−0.28 (0.12)	0.142	−0.56 (0.36)	0.174
Group 3				
Physical activity	−0.4 (0.16)	0.086	−0.1 (0.37)	0.798
Lipid lowering	−0.15 (0.09)	0.182	−0.65 (0.39)	0.138

Abbreviations: HMGCR, 3-hydroxy-3-methylglutaryl-CoA, reductase; LDL, low-density lipoprotein cholesterol; PCSK9, proprotein convertase subtilisin/kexin type 9 serine protease. Footnote: In the multivariate Mendelian randomization analysis, we divided the factors into three groups: lifestyle factors (smoking status and alcohol intake frequency), dietary intake (selenium and iron), and accelerometer-based physical activity measurement, and these factors were included in the analysis one group at a time.

## Discussion

Our drug-target MR study showed that inhibition of the PCSK9 gene was causally associated with a lower risk of SLE in the European population, but this association was not confirmed in the East Asian population, suggesting the diversity in the causal effect of PCSK9 inhibition on SLE risk in different populations. Inhibition of the HMGCR gene (mimicking the effect of statins) and NPC1L1 (mimicking the effect of ezetimibe) were not associated with SLE risk in either the European or East Asian populations. Secondary and sensitivity analyses supported the findings of the primary IVW analysis. Stratification analysis showed that the effect of PCSK9 inhibition on SLE risk reduction was similar in male and female populations, suggesting that sex stratification did not affect the effect. Our study also found that the risk of SLE was independent of serum LDL cholesterol levels, suggesting that the effect of PCSK9 inhibition may not be the result of lipid downregulation. Multivariate analysis showed that intake of iron and physical activity mediated a part of the PCSK9 inhibition effect on SLE risk.

There is a lack of evidence from RCTs that support the benefit of lipid-lowering drugs for the reduction of SLE risk. The result of our study could be treated as evidence from a quasi-RCT, which provides stronger evidence than those from traditional observational studies, and this is one strength of the study. In addition, we applied data from differential study populations (i.e., European and East Asian populations), which promoted the discovery that inhibition of PCSK9 had diverse effects on SLE risk in the differential populations.

Research on the impact of lipid-lowering drugs on the risk of skin diseases has been conducted over the past 5 years. A recent MR study published by Zhao et al. (2023) found that serum LDL levels did not correlate with the risk of psoriasis (Zhao et al., 2023). However, when simulating the lipid-lowering effect using genetic instruments, diverse effects on psoriasis risk were observed. Inhibition of PCSK9 was associated with a lower risk of psoriasis, whereas inhibition of HMGCR and NPC1L1 did not have an impact. Our MR study yielded similar results, showing that only the inhibition of PCSK9 reduced the risk of SLE. These findings are consistent with observational studies conducted over the past 5 years. For instance, a study by Sánchez-Pérez et al. (2020) reported that SLE patients with higher disease activity and more severe lesions had elevated serum levels of PCSK9. It was reported that SLE patients with higher disease activity and more severe lesions had higher serum PCSK9 levels. The association between higher PCSK9 levels and more severe SLE disease activity was further confirmed by another study from Karolinska University Hospital (Liu et al., 2020). In addition, the elevated PCSK9 levels were also found to correlate with atherogenic inflammation in SLE (Fang et al., 2018), which caused a higher rate of accelerated atherosclerosis—a pathological factor contributing to cardiovascular disease. These studies all suggested the benefit of inhibiting PCSK9 function in SLE patients. Interestingly, this effect seems to only exist in PCSK9 inhibition, but not in other lipid-lowering drug targets (i.e., HMGCR inhibition). For example, although inhibition of PCSK9 was causally associated with lower LDL cholesterol levels and lower risk of atherogenesis in SLE patients (Fang et al., 2018), statins had no benefit for atherogenesis in SLE patients (Schanberg et al., 2012; van Leuven et al., 2012). Combining the results of previously published studies and those of our study, we hypothesized that evolocumab (the PCSK9 inhibitor) would be effective in reducing the severity of SLE or in preventing SLE.

Although previous studies and our study suggest a protective effect of PCSK9 inhibition on SLE risk, prospective studies are still needed to verify the effect. Three aspects need to be focused on. First, the protective effect of PCSK9 inhibition on atherosclerosis in patients with SLE should be evaluated because atherosclerosis is highly prevalent in SLE patients and PCSK9 inhibition has been suggested to be beneficial for this condition. Second, the adjuvant effect of PCSK9 inhibition in the treatment of SLE should be evaluated, especially in those with elevated LDL-C levels. PCSK9 inhibition may reduce the severity of SLE (Liu et al., 2020) and lower LDL-C levels to prevent cardiovascular events in SLE patients. Third, the long-term effect of PCSK9 on SLE risk has not been studied, which may be addressed by an observational cohort study evaluating the efficacy of evolocumab (the PCSK9 inhibitor) on SLE severity and complications in one to 5 years after evolocumab initiation.

The mechanism underlying the association between PCSK9 inhibition and reduced SLE risk remains unclear. However, based on current study findings, several hypotheses can be proposed. First, this effect is a result of inflammation suppression. Previous research has indicated that PCSK9 can induce the secretion of pro-inflammatory cytokines in different types of tissues and modulate the expression of toll-like receptor 4 and activation of NF-κB (Ding et al., 2020). As a result, the inhibition of PCSK9 can alleviate inflammation pathways, leading to a decreased risk of SLE—an autoimmune disease characterized by inflammation affecting various organs. For instance, renal damage is a prominent manifestation of SLE, and lupus nephritis occurs when immune complexes deposit in the kidney glomeruli. The treatment of lupus nephritis typically involves immunosuppressive therapy (Anders et al., 2020). Second, the effect of PCSK9 inhibition on reducing SLE risk could be attributed to the interplay between PCSK9 and oxidized LDL function. Previous studies have shown that oxidized LDL can stimulate the activation of dendritic cells, and this process is dependent on the function of the PCSK9 gene. Additionally, a study identified a correlation between PCSK9 function and the severity of SLE, suggesting an interaction between oxidized LDL and PCSK9 (Liu et al., 2020). Another study further demonstrated an interaction of PCSK9 and receptor 1 of oxidized LDL (Ding et al., 2020). Oxidized LDL has been shown to have a proinflammatory effect and can induce the immune activation of monocytes and T cells (Huang et al., 1995). The inhibition of PCSK9 can improve OxLDL-induced immune activation and inhibit the activation and maturation of dendritic cells. Moreover, PCSK9 inhibition has been found to promote an increase in T regulatory cells, which are thought to have a protective effect in SLE. Third, PCSK9 inhibition might work by inhibiting macrophage activation and pro-inflammatory response. It was found that PCSK9 induced macrophage activation and pro-inflammatory response (Ricci et al., 2018; Katsuki et al., 2022). The imbalance in the polarization and the abnormal activation of the macrophages are closely related to the onset and the development of SLE. Fourth, it might work by platelet-activating factor (PAF) and its precursors. PAF has been shown to induce enhanced interferon-gamma (IFN-gamma) secretion from peripheral blood mononuclear cells (PBMCs) (Frostegård et al., 1997). The PAF and PAF-like lipids in OxLDL also significantly activate immunoreactive cells to produce IFN-gamma. In patients with SLE, increased expression of IFN marker genes is associated with severe organ damage (such as nephritis and central nervous system damage) and disease activity. Therefore, inhibition of PCSK9 may also be beneficial in SLE through this mechanism and not directly related to its lipid-lowering ability.

Our study results provided several implications for clinical practice and future studies on SLE prevention and treatment. Although SLE often affects women of child-bearing age, approximately 10%–20% of the cases had elderly-onset lupus, and menopause and cellular immunity changes were the major contributors for elderly-onset SLE (Lazaro, 2007). Regarding the lack of treatment modalities and studies for the elderly-onset SLE (Rovenský and Tuchynová, 2008; Fettig et al., 2013), our study could serve as *prima facie* evidence to guide adding PCSK9 inhibitors (i.e., evolocumab) for the elder population with a high risk of SLE, especially those accompanied with abnormally high levels of LDL cholesterol. Before putting it into practice, studies are warranted to clarify whether PCSK9 inhibitors were efficacious in lowering SLE risk and disease severity, and the mechanism of their effect. RCT might be needed for clarifying the specific effect of PCSK9 inhibitors for SLE. Mechanism studies might focus on the suppression of autoimmune-related inflammation.

Our study had limitations similar to previously published MR studies. First, we adopted a two-sample MR design and utilized summary-level data, which made adjustment for important covariates impossible. For example, the severity of SLE may be an important covariate that affects our study results, since one study reported that the severity of SLE was associated with serum levels of PCSK9 (Liu et al., 2020). We performed stratification analysis to imitigate the impact of this limitation, which showed that sex had no impact on the effect of PCSK9 inhibitors on SLE risk. However, to further investigate the causal relationship between PCSK9 inhibitors and SLE risk prevention, future studies should collect data at the individual participant level. Second, although the MR design was less prone to confounding issues, it could be affected by pleiotropic effects. We used MR-Egger and MR-PRESSO methods to detect the pleiotropic effects, and we used multivariate MR to discover important effect mediators. For example, we found that physical activity mediated part of the effect of PCSK9 inhibition on SLE risk, which could be explained by the fact that patients taking lipid-lowering drugs were more likely to engage in physical activity to help lower lipid levels. However, the statistical methods could not exclude the possibility that the preventive effect on SLE risk was caused by PCSK9 inhibition. Conducting a randomized controlled trial to test the efficacy of PCSK9 inhibitors in preventing SLE and reducing SLE severity would be a better option for future studies. Third, the MR design assesses the lifetime effect of an exposure on an outcome, which suggested the probability of overestimation of the PCSK9 inhibition effect. Therefore, prospective RCTs are warranted to test the actual effect in clinical settings. Fourth, the study predominantly focuses on European and East Asian populations, leaving out other ethnic populations. The generalizability of the study results should be further evaluated. Future studies should consider including diverse ethnic groups and races to validate the findings across a broader population spectrum and promote global reliability.

## Conclusion

Our MR study demonstrated a beneficial effect of PCSK9 inhibition on lowering SLE risk in the European population. This protective effect was not replicated in the East Asian population and was not affected by sex. No significant protective effects were observed for HMGCR and NPC1L1. These fingdings suggested that evolocumab—the PCSK9 inhibitor—might exert protective effects on SLE, but statins and ezetimibe might not have the protective effect. Further research is necessary to elucidate the specific mechanisms and potential therapeutic applications of evolocumab in the context of SLE protection.

## Data Availability

The original contributions presented in the study are included in the article/Supplementary Material, further inquiries can be directed to the corresponding authors.
