# Supplementary material for: Association of lipid lowering drugs and the risk of systemic lupus erythematosus: a drug target Mendelian randomization
Source: Front Pharmacol. 2023 Oct 30;14:1258018. doi: 10.3389/fphar.2023.1258018 (PMC10642506; doi:10.3389/fphar.2023.1258018)
Supplement: Supplementary file 1 [file Table1.docx]

**Outline**

[Supplementary tables 1. The selected SNPs for predicting lipid-lowering drugs 2](#_Toc147003466)

[Supplementary Table 2. The secondary analyses 4](#_Toc147003467)

[Supplementary Table 3. Stratification analysis 6](#_Toc147003468)

[Supplmentary Table 4. MR Egger regression analysis 8](#_Toc147003469)

[Supplementary Figure 1. Colocalization analysis for HMGCR gene inhibition and risk of SLE 9](#_Toc147003470)

[Supplementary Figure 2. Colocalization analysis for PCSK9 gene inhibition and risk of SLE 10](#_Toc147003471)

[Supplementary Figure 3. Colocalization analysis for NPC1L1 gene inhibition and risk of SLE 11](#_Toc147003472)

## Supplementary tables 1. The selected SNPs for predicting lipid-lowering drugs

| **SNP** | **Position**  **(Build 37)** | **Non-effect allele** | **Effect allele frequency** | **Effect (95% CI), mmol/L** | **P-value** | **F-statistic** |
| --- | --- | --- | --- | --- | --- | --- |
| **HMGCR** |  |  |  |  |  |  |
| rs12916 | chr5:74656539 | C | 0.57 | -0.073 (-0.081, -0.066) | 7.79E-78 | 349.14 |
| rs12173076 | chr5:74697050 | G | 0.88 | -0.065 (-0.076, -0.054) | 2.33E-27 | 117.45 |
| rs10515198 | chr5:74641560 | A | 0.9 | -0.060 (-0.072, -0.048) | 5.99E-22 | 92.75 |
| rs3857388 | chr5:74620377 | C | 0.87 | -0.042 (-0.054, -0.031) | 2.2E-11 | 44.78 |
| rs7711235 | chr5:74540397 | G | 0.73 | -0.038 (-0.050, -0.025) | 5E-10 | 38.68 |
| **PCSK9** |  |  |  |  |  |  |
| rs11591147 | chr1:55505647 | G | 0.01715 | -0.497 (-0.532, -0.462) | 8.6E-143 | 650.03 |
| rs11206510 | chr1:55496039 | T | 0.1544 | -0.083 (-0.093, -0.073) | 2.38E-53 | 236.57 |
| rs2479409 | chr1:55504650 | G | 0.6675 | -0.064 (-0.072, -0.056) | 2.51E-50 | 222.69 |
| rs585131 | chr1:55524116 | T | 0.18 | -0.064 (-0.074, -0.054) | 2.7E-35 | 153.76 |
| rs11206514 | chr1:55516004 | A | 0.39 | -0.051 (-0.059, -0.043) | 9.95E-33 | 142.01 |
| rs2495477 | chr1:55518467 | A | 0.4 | -0.064 (-0.075, -0.053) | 7.28E-30 | 128.96 |
| rs572512 | chr1:55517344 | T | 0.65 | -0.048 (-0.057, -0.039) | 5.31E-26 | 111.25 |
| rs2479394 | chr1:55486064 | G | 0.715 | -0.039 (-0.047, -0.031) | 1.58E-19 | 81.71 |
| rs12067569 | chr1:55528629 | A | 0.97 | -0.089 (-0.108, -0.069) | 1.97E-17 | 72.19 |
| rs10493176 | chr1:55538552 | T | 0.1148 | -0.078 (-0.098, -0.058) | 2.54E-14 | 58.08 |
| rs11583974 | chr1:55551718 | A | 0.97 | -0.065 (-0.088, -0.042) | 3.95E-09 | 34.65 |
| **NPC1L1** |  |  |  |  |  |  |
| rs2073547 | chr7:44582331 | G | 0.81 | -0.049 (-0.058, -0.039) | 1.92E-21 | 90.44 |
| rs217386 | chr7:44600695 | G | 0.41 | -0.036 (-0.044, -0.029) | 1.2E-19 | 82.26 |
| rs7791240 | chr7:44602589 | C | 0.91 | -0.043 (-0.057, -0.029) | 1.84E-10 | 41 |

**Abbreviations:** HMGCR, 3-hydroxy-3-methylglutaryl-CoA reductase. LDL, low-density lipoprotein cholesterol. NPC1L1, Niemann-Pick C1-Like 1. PCSK9, proprotein convertase subtilisin/kexin type 9 serine protease. SNP, single nucleotide polymorphism. SLE, systemic lupus erythematosus.

## Supplementary Table 2. The secondary analyses

| **Exposure** | **Population** | **Method** | **No. of SNP** | **Beta** | **Se** | **P-value** |
| --- | --- | --- | --- | --- | --- | --- |
| HMGCR | European | IVW | 5 | -0.227 | 0.284 | 0.424 |
| HMGCR | European | MR Egger | 5 | -0.373 | 0.870 | 0.697 |
| HMGCR | European | Simple mode | 5 | -0.201 | 0.448 | 0.676 |
| HMGCR | European | Weighted median | 5 | -0.248 | 0.296 | 0.402 |
| HMGCR | European | Weighted mode | 5 | -0.260 | 0.338 | 0.483 |
| HMGCR | East Asian | IVW | 2 | -0.128 | 0.309 | 0.679 |
| PCSK9 | European | IVW | 10 | -0.678 | 0.207 | 0.001 |
| PCSK9 | European | MR Egger | 10 | -0.679 | 0.336 | 0.078 |
| PCSK9 | European | Simple mode | 10 | -1.002 | 0.386 | 0.029 |
| PCSK9 | European | Weighted median | 10 | -0.788 | 0.242 | 0.001 |
| PCSK9 | European | Weighted mode | 10 | -0.863 | 0.227 | 0.004 |
| PCSK9 | East Asian | IVW | 2 | 0.051 | 0.526 | 0.923 |
| NPC1L1 | European | IVW | 2 | -0.546 | 1.452 | 0.706 |
| LDL | European | IVW | 301 | -0.049 | 0.115 | 0.671 |
| LDL | European | MR Egger | 301 | -0.142 | 0.178 | 0.425 |
| LDL | European | Simple mode | 301 | 0.815 | 0.328 | 0.013 |
| LDL | European | Weighted median | 301 | -0.227 | 0.146 | 0.119 |
| LDL | European | Weighted mode | 301 | -0.335 | 0.174 | 0.055 |
| LDL | East Asian | IVW | 30 | -0.159 | 0.171 | 0.35 |
| LDL | East Asian | MR Egger | 30 | -0.021 | 0.274 | 0.938 |
| LDL | East Asian | Simple mode | 30 | 0.056 | 0.216 | 0.795 |
| LDL | East Asian | Weighted median | 30 | -0.093 | 0.123 | 0.449 |
| LDL | East Asian | Weighted mode | 30 | -0.079 | 0.117 | 0.503 |

**Abbreviations:** HMGCR, 3-hydroxy-3-methylglutaryl-CoA reductase. IVW, inverse variance weighted. LDL, low-density lipoprotein cholesterol. NPC1L1, Niemann-Pick C1-Like 1. PCSK9, proprotein convertase subtilisin/kexin type 9 serine protease. SNP, single nucleotide polymorphism. SLE, systemic lupus erythematosus.

## **Supplementary Table** 3**. Stratification analysis**

| **Exposure** | **Population** | **Method** | **No. of SNP** | **Beta** | **Se** | **P-value** |
| --- | --- | --- | --- | --- | --- | --- |
| HMGCR | Female | IVW | 5 | -0.210 | 0.259 | 0.417 |
| HMGCR | Female | MR Egger | 5 | -0.337 | 0.745 | 0.682 |
| HMGCR | Female | Simple mode | 5 | -0.179 | 0.395 | 0.674 |
| HMGCR | Female | Weighted median | 5 | -0.222 | 0.286 | 0.439 |
| HMGCR | Female | Weighted mode | 5 | -0.235 | 0.284 | 0.455 |
| HMGCR | Male | IVW | 5 | -0.228 | 0.291 | 0.433 |
| HMGCR | Male | MR Egger | 5 | -0.375 | 0.958 | 0.722 |
| HMGCR | Male | Simple mode | 5 | -0.209 | 0.455 | 0.669 |
| HMGCR | Male | Weighted median | 5 | -0.258 | 0.305 | 0.398 |
| HMGCR | Male | Weighted mode | 5 | -0.273 | 0.322 | 0.444 |
| PCSK9 | Female | IVW | 10 | -0.683 | 0.211 | 0.001 |
| PCSK9 | Female | MR Egger | 10 | -0.647 | 0.323 | 0.080 |
| PCSK9 | Female | Simple mode | 10 | -1.119 | 0.362 | 0.013 |
| PCSK9 | Female | Weighted median | 10 | -0.787 | 0.227 | 0.001 |
| PCSK9 | Female | Weighted mode | 10 | -0.860 | 0.255 | 0.008 |
| PCSK9 | Male | IVW | 10 | -0.654 | 0.197 | 0.001 |
| PCSK9 | Male | MR Egger | 10 | -0.692 | 0.332 | 0.071 |
| PCSK9 | Male | Simple mode | 10 | -0.907 | 0.326 | 0.021 |
| PCSK9 | Male | Weighted median | 10 | -0.771 | 0.225 | 0.001 |
| PCSK9 | Male | Weighted mode | 10 | -0.832 | 0.246 | 0.008 |
| NPC1L1 | Female | IVW | 2 | -0.556 | 1.588 | 0.726 |
| NPC1L1 | Male | IVW | 2 | -0.465 | 1.214 | 0.702 |
| LDL | Female | IVW | 220 | -0.022 | 0.096 | 0.821 |
| LDL | Female | MR Egger | 220 | -0.153 | 0.143 | 0.287 |
| LDL | Female | Simple mode | 220 | -0.876 | 0.310 | 0.005 |
| LDL | Female | Weighted median | 220 | -0.106 | 0.137 | 0.439 |
| LDL | Female | Weighted mode | 220 | -0.187 | 0.141 | 0.187 |
| LDL | Male | IVW | 213 | 0.057 | 0.128 | 0.655 |
| LDL | Male | MR Egger | 213 | -0.030 | 0.204 | 0.883 |
| LDL | Male | Simple mode | 213 | 0.769 | 0.291 | 0.009 |
| LDL | Male | Weighted median | 213 | -0.123 | 0.146 | 0.399 |
| LDL | Male | Weighted mode | 213 | -0.095 | 0.145 | 0.514 |

**Abbreviations:** HMGCR, 3-hydroxy-3-methylglutaryl-CoA reductase. IVW, inverse variance weighted. LDL, low-density lipoprotein cholesterol. NPC1L1, Niemann-Pick C1-Like 1. PCSK9, proprotein convertase subtilisin/kexin type 9 serine protease. SNP, single nucleotide polymorphism. SLE, systemic lupus erythematosus.

## Supplmentary Table 4. MR Egger regression analysis

| **Exposure** | **Population** | **Estimate of horizontal pleiotropy (intercept)** | **Standard error of intercept** | **p-value of intercept** | **Q statistics** |
| --- | --- | --- | --- | --- | --- |
| HMGCR | European | 0.009 | 0.05 | 0.871 | 0.747 |
| PCSK9 | European | 0.00009 | 0.02 | 0.996 | 11.17 |
| NPC1L1 | European | 0.033 | 0.07 | 0.704 | 0.636 |
| LDL | European | 0.002 | 0.004 | 0.659 | 677.2 |
| LDL* | East Asian | -0.006 | 0.016 | 0.71 | 118.4 |

**Abbreviations:** HMGCR, 3-hydroxy-3-methylglutaryl-CoA reductase. IVW, inverse variance weighted. LDL, low-density lipoprotein cholesterol. NPC1L1, Niemann-Pick C1-Like 1. PCSK9, proprotein convertase subtilisin/kexin type 9 serine protease.

**Footnote:** The intercept term in MR Egger regression can be a useful indication of whether directional horizontal pleiotropy is driving the results of an MR analysis. We reported the intercept values and corresponding p-values for the MR Egger analysis, and we found no evidence of pleiotropic effect in these analyses.

* Owing to the small number of SNPs screened in the East Asian population, the MR Egger intercept values could not be calculated for HMGCR, PCSK9, and NPC1L1.

## Supplementary Figure 1. Colocalization analysis for HMGCR gene inhibition and risk of SLE


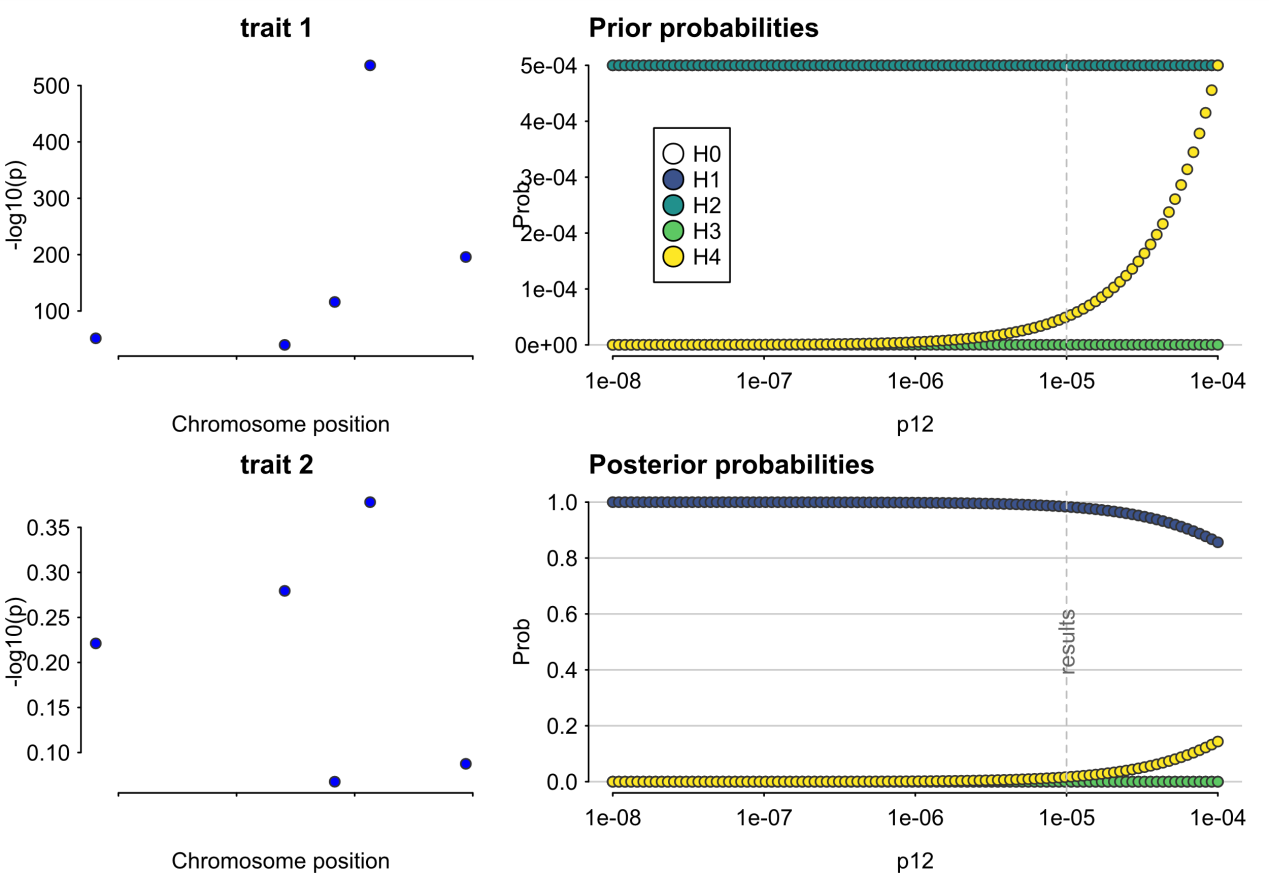


**Abbreviations:** HMGCR, 3-hydroxy-3-methylglutaryl-CoA reductase. SLE, systemic lupus erythematosus.

**Footnotes:** The sensitivity analysis was performed with the passing rule of 0.9. The posterior probability of for shared variant was 1.65%.

## Supplementary Figure 2. Colocalization analysis for PCSK9 gene inhibition and risk of SLE


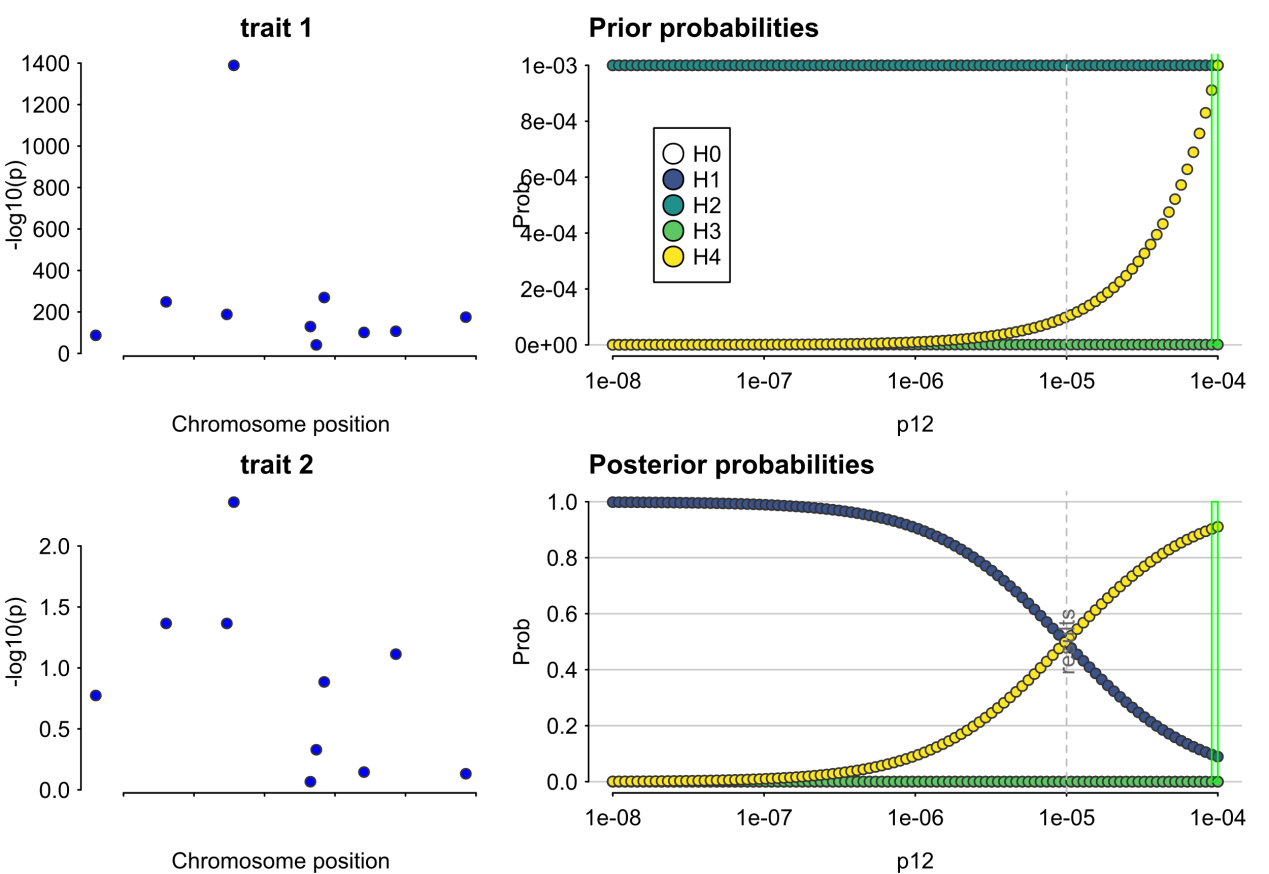


**Abbreviations:** PCSK9, proprotein convertase subtilisin/kexin type 9 serine protease. SLE, systemic lupus erythematosus.

**Footnotes:** The sensitivity analysis was performed with the passing rule of 0.9. The posterior probability of for shared variant was 50.4%.

## Supplementary Figure 3. Colocalization analysis for NPC1L1 gene inhibition and risk of SLE


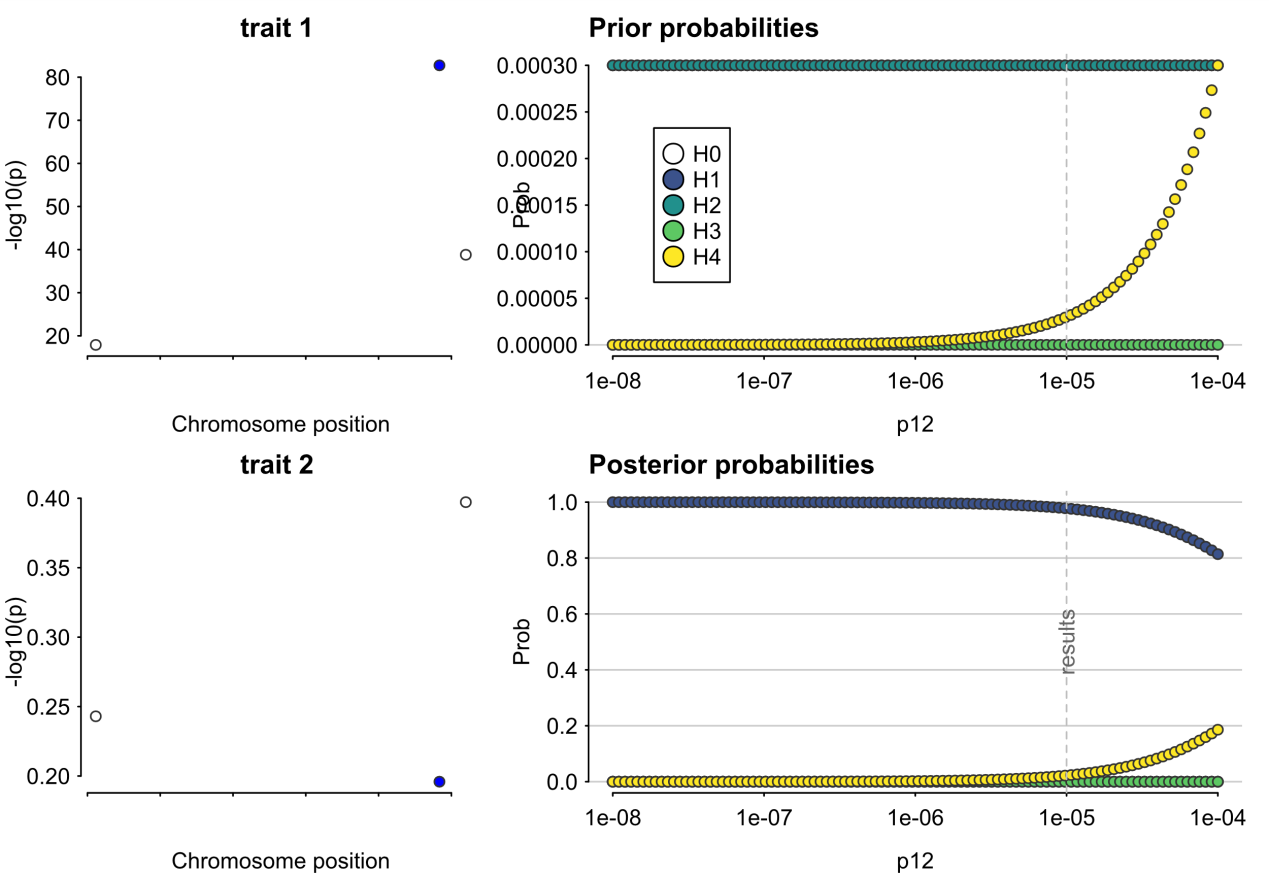


**Abbreviations:** NPC1L1, Niemann-Pick C1-Like 1. SLE, systemic lupus erythematosus.

**Footnotes:** The sensitivity analysis was performed with the passing rule of 0.9. The posterior probability of for shared variant was 2.23%.
